# Spatial Variability of Health Inequalities of Older People in China and Related Health Factors

**DOI:** 10.3390/ijerph17051739

**Published:** 2020-03-07

**Authors:** Mengqi Yang, Mark W. Rosenberg, Jie Li

**Affiliations:** 1 School of Geographical Sciences, Guangzhou University, Guangzhou 510006, China; yangmengqi0988@gmail.com; 2 Department of Geography and Urban Planning, Queen’s University, Kingston, ON K7L 3N6, Canada; mark.rosenberg@queensu.ca; 3 College of Resources and Environmental Science, Ningxia University, Yinchuan 750021, China

**Keywords:** health inequalities, life expectancy, older people, spatial distribution, socio-economic factor, Moran’s I, Getis-Ord Gi*

## Abstract

China is facing serious population aging issues because of many unintended consequences of the economic reforms that began in the 1980s and with social policies such as the “one child” policy. Understanding the spatial distribution of the health status of older people has attracted more and more attention in many countries, including China. By employing descriptive analysis, this study uses data from the Chinese Population Censusand Statistical Year Bookto explore the health inequalities of older people at the national level. Based on the Getis-Ord Gi*, this study finds that the uneven spatial distribution of socio-economic status results in health inequalities for older people at the national level. The geographic distribution of life expectancy was correlated with a number of important demographic, socio-economic, and environmental variables. For further research, investigations should be conducted among individuals at micro-geographic scales.

## 1. Introduction

Population aging spawns many significant influences in a society. Understanding the role of the aging process has become an important issue for both politicians and scholars all around the world. Many variables can be used to explain health inequalities and it can also operate at various geographic scales. 

The increase in the older population is normally a symbol of living standards and health care improvements. However, different countries exhibit different patterns. Take China as an example. Over 38 years, from 1980 to 2018, the life expectancy in China increased by 15.3 percent. When compared with 1950, life expectancy increased by 90.3 percent [1]. The dramatic increase of life expectancy has been driven by three aspects: (1) rapid economic growth, leading to improvement of living standards and wealth accumulation; (2) the “baby boom” in 1950s and the “one child” policy in 1980s, leading to the growth of older people and the decrease of fertility rate; (3) furthermore, the improvement in health and longevity of people in China, which varies greatly by regions. The longest life expectancy in Shanghai is 80.26 years old, while the number is 68.17 in Xizang [2,3], the geographic disparities among provinces result in health inequalities in China. Developed countries are ”getting older after getting rich”, whereas China is “getting older while getting rich” [4]. With the negative conditions of poverty and inadequate health care resources, China is encountering intense population pressures, which can lead to social and financial burdens for people and influence the sustainability of society. 

Research suggests that people’s demographic characteristics contribute to the improvement of life expectancy [5]. Gender interacts with health on many dimensions that produce different outcomes with respect to physical and social contact. The most well-known knowledge is that women live longer than men, while they are likely to suffer from elevated morbidity rates. Research shows that people with lower educational levels report worse health and are likely to die earlier than well-educated people [6]; it is because well-educated people will have better financial status and they are likely to have a better sense of self-control compared to less educated people. Also, the relationship between socioeconomic status (SES) and health is one of the most widely reported phenomena in the health literature. It is well recognized that people with higher SES are healthier than people in lower SES [7]. Generally, there is a positive correlation between income and health outcomes [8]. A considerable body of income-health related research suggested that better health favors higher-income groups [9,10,11]. Increasing income inequality affects the health status of the population overall [12]. The adverse health impact of income is even worse for older people than for younger groups [13]. Other characteristics addressing SES include martial status and living arrangements. It has been demonstrated that being divorced or widowed is associated with poorer health because of marriage protection and selection effects [14]. As people grow older, they tend to benefit more from the impacts of marriage. Married adults are healthier than their unmarried counterparts. One of the reasons is that marriage financially strengthens family capacity; another mechanism is the marriage selection effect that healthier individuals are more likely to marry and stay married [15]. An international comparison on life course adversity and physical performance of older people in four countries showed that older people living alone are associated with low physical performance [16]. People living alone are associated with worse health outcomes. More research has been conducted to gain more insights into health inequalities as a function of access to health resources [17]. People, who are able to access well-equipped community or health care centres are likely to report better health outcomes [18,19,20,21,22]. Health inequalities not only occur between different countries but also occur for population groups within a country. 

Most research on health inequalities has been carried out in English-speaking countries. Because of the shorter period of economic development and the rapid aging process, research on health inequalities is relatively limited in China and there is even less research on older people. For instance, gender and health inequalities research is conducted with an assumption that individuals are situated within certain social, cultural, and political contexts. The health advantage or disadvantage for females that can be characterized in western industrial countries does not necessarily apply to other countries. In China, due to the increased population in baby booming in 1950s, and the long history of advocacy for the rights of males over females in Chinese culture, it is interesting to explore if health inequalities are more or less marked between females and males among the older population. With regard to the key virtues of filial piety and dutiful payment, it suggests that older parents should be taken care of by their adult children, and live with their children. Due to the rapid urbanization process and the migration of the labor force to cities, parents cannot live with their children, but the influence of the living arrangements on older people’s health is changed and unknown. What is more, unlike western countries, the Chinese household registration system is a dual system. Within this system, urban residents enjoy a range of social, economic, and cultural benefits, whereas rural residents do not receive the same benefits [14]. This system is closely associated with the social security system that excludes rural residents/migrant workers from the city-wide social security system [23]. Despite the remarkable research in health outcomes over the past decades, there is a limited amount of research on health inequalities in China, which deserves more attention. Chinese health inequalities have their own characteristics, where a comprehensive study is urgently needed. It is necessary to find out whether the well-established health inequality assumptions work in the same way in China or not. 

Based on the date from the Chinese Statistical Year Book 2018, spatial analysis was applied to identify the spatial clustering of life expectancy and to describe the geographic distribution of health inequalities in China. In order to investigate the determinants of health inequalities of older people in China, demographic and SES variables were selected from the 2010 Population Census of the People's Republic of China and the China Statistical Year Book of Environment 2010. Correlation analysis was conducted to study the relationships between older people's health status and health-related variables.

## 2. Materials and Methods

This study is a descriptive analysis at the national level in China. Data for this study come from the 2010 Population Census of the People’s Republic of China [1], the Chinese Statistical Year Book 2018 [2], and the China Statistical Year Book of Environment 2010 [3].These national-level statistics cover all people residing in the territory of the People’s Republic of China, including 31 provinces, autonomous regions and municipalities that have data. We do not have data for Hong Kong, Macau, and Taiwan, therefore, they are excluded from the study. 

The general file is made up of questions that reflect the population’s demographic characteristics, SES, health care utilization characteristics, as well as people’s geographic characteristics. Table 1 summarizes all variables selected from the previous three datasets. 

Based on the date from the Chinese Statistical Year Book 2018, spatial analysis was applied to identify the spatial clustering of life expectancy and to describe the geographic distribution of health inequalities in China. Life expectancy was selected form the Chinese Statistical Year Book is an annual statistical, which reflects comprehensively the economic and social development of China. It covers the key statistical data for the local levels of province in 2018. The use of the latest date of the Chinese Statistical Year Book 2018 helps us understand the current situation of life expectancy of older people in China.

In this research, life expectancy is chosen as the dependent variable measured as the average number of years of life expectancy for both females and males in each province (31 provinces in total). Life expectancy is understood as “the expected number of years to be lived, on the average, by a particular population at a particular time” [24]. For a more detailed understanding of how life expectancy is calculated. 

In order to investigate the determinants of health inequalities of older people in China, demographic and SES variables were selected from the 2010 Population Census of the People's Republic of China and the China Statistical Year Book of Environment 2010. China conducted the 6th national population census with the participation of 1.3 billion people of all nationalities. The 2010 population census covers all people residing in the territory of the People’s Republic of China and the Chinese citizens residing outside but not permanently settled down in locations beyond the territory of the People’s Republic of China at the census reference time, excluding residents of Hong Kong, Macao and Taiwan and foreigners temporarily staying in the territory of the People’s Republic of China. Territory here refers to the territory of the customs. Two types of questionnaires were used for the 2010 population census. The short form contains items that reflect the basic situation of the population, while the long form includes all short form items plus other items, such as economic activities, marriage and family, fertility and housing.

The China Statistical Yearbook on Environment 2010 was prepared jointly by the National Bureau of Statistics, Ministry of Ecology and Environment and other ministries. It is an annual statistics publication, with comprehensive data in 2010 and selected data series in major years at the national and provincial levels, reflecting various aspects of China’s environmental development.

Variables that reflect older people’s demographic characteristics and SES are selected form those two databases. Correlation analysis was conducted to study the relationships between older people's health status and health-related variables. Because this research is a secondary analysis of data by the National Bureau of Statistics, which is available to the public without identifiable information, an ethics review of the data collection method is therefore not required. The date can be found at http://www.stats.gov.cn/tjsj/ndsj/ . The 6th national population census was conducted in 2010. In order to match the census data, we selected the China Statistical Yearbook on Environment for the year 2010.

In terms of demographic characteristics of older population, the percentage of older people indicates the population distribution of older people to the total population in each province. The percentage of older old (people aged 80 years old and older) and younger old (people aged 60 to 79 years old) reflects the age distribution of older people (60 and older) in each province. With respect to gender, the percentage of female older persons and the percentage of male older persons show the gender distribution in each province. It is the ratio between female/male older people and total population in each province.

Two variables are used to study older people’s income status: income inequalities and the percentage of people who live on minimum living allowances. The ratio between older people with high income and low income is defined as a general estimate of income inequalities in each province. The degree of income inequalities reflects if the income is equally distributed among older people. The increased income inequality affects the health status of the population negatively. 

The percentage of people who live on minimum living allowances is a variable of the distribution of absolute wealth of older people in each province. The old dependency ratio is calculated as the ratio between the number of older people and the number of working-age people of each geographic unit. It is used to reflect the relative financial support for older people. A high dependency ratio represents people of working age who face a greater burden in supporting the aging population, which can be shifted to reflect the increased needs associated with older people.

Older people’s educational status is measured by the percentage of illiterate people (i.e., older people who cannot read) in each province. The ratio between older people who cannot read and older population in each province.

In this research, marital status is measured by two indicators. The percentage of divorced and widowed is the ratio between divorced or windowed older people and total older population in each province. Unlike western countries, being single is not quite the fashion in Chinese culture, especially for older people. The amount of unmarried older people is very small. For this reason, we do not have a particular variable for single older people. The percentage of people with married status is the ratio between married older people and total older population. 

Regarding the living arrangements for older people, the ratio between one person and more is used to describe if older people live alone or not. It is the ratio between older people who live alone or live with more than one person. In addition, the ratio between older people living along and living with more than one person is used to describe if older people are physically isolated from their children. 

The percentage of hospitals and health care centers are variables that reflect health care accessibility in each province. It is the ratio between the number of hospitals in each province to the total number of hospitals in China. The number of health care centers per 1000 population is used as a measure of health care accessibility in each province. It is the ratio between the number of health care centers in each province to the number of health care centers in China. For most older people in China, they tend to stay home and are taken care by their families, in this case, hospitals and health care centers are only places where older people can get professional medical assistance; also, we do not have the data for the number of people who visit doctors or take medicine. The percentage of hospitals and health care centers and the percentage of health care centers per 1000 population are the most suitable variables to reflect the situation of the health care access that older people have in this research.

The percentage of non-agricultural *Hukou* is an important variable to reflect the geographic distribution of the older population in each province. It is the ratio between people with non-agriculture *Hukou* to agriculture *Hukou* in each province. Due to the lack of statistical data of older people’s *Hukou* status, in this research, we used the *Hukou* status in each province to represent the older people’s *Hukou* status. The Chinese household registration system is closely associated with the social security system that excludes rural residents. It is difficult for people with agriculture *Hukou* to change their *Hukou*. For this reason, older people who live in urban areas may still keep their agriculture *Hukou*. We use the variable *Hukou* status in each province only to give a brief presentation for older people’s *Hukou* status.

This study is conducted following a hypothesis that there are statistically significant relationships between health inequalities of older people and health-related variables in China. Sub-hypotheses include the more older people are, the more income inequalities are negatively related to life expectancy; being married is positively related to health outcomes of older people (being divorced or widowed has negative influence on older people’s life expectancy); a better health environment helps to narrow health inequalities; and people with urban *Hukou* report better health outcomes than rural people.

Statistical analyses are conducted using SPSS Version22 (SPSS, Inc., Chicago, IL, USA) and ArcGIS version 10.7. (ESRI, RedLands, CA, USA) Skewness and kurtosis were visually examined against normal distribution and a quantile–quantile plot was drawn against the normal curve. The result showed that all the variables are normally distributed. All the variables to check if all variables are normally distributed. Pearson’s correlation is used to measure the degree of the linear correlation between variables. 

Global Moran’s I is a measure of spatial autocorrelation based on both the locations of the variables and attribute values simultaneously; it evaluates whether the pattern expressed is clustered, dispersed, or random. The value of Moran’s I ranges from −1 to 1. The closer Moran’s I is to 1, the higher the spatial correlation; the closer Moran’s I is to −1, the greater the spatial disparity. In our study, global Moran’s I is used to evaluate the pattern of spatial clustering of health inequalities of older people in China. 

Mathematically, Moran's I is expressed as

(1) $$I=\frac{n}{S_{0}}\frac{\sum_{i=1}^{n}\sum_{j=1}^{n}w_{i,j}z_{i}z_{j}}{\sum_{i=1}^{n}z_{i}^{2}}$$

where $z_{i}$ is the deviation of an attribute for feature i from its mean $\left(x_{i}-\;\bar{X}\right)$, $w_{i,j}$ is the spatial weight between features i and j, *n* is the total number of features, and $S_{0}$ is the aggregate of all the spatial weights.

(2) $$S_{0}=\overset{n}{\underset{i=1}{\sum }}\overset{n}{\underset{j=1}{\sum }}w_{i,j}$$

The results of the global Moran's I analysis are interpreted within the context of its null hypothesis of random spatial distribution. When the *p*-value is not statistically significant, one cannot reject the null hypothesis. When the *p*-value is statistically significant, then the spatial distribution is not randomly distributed. A positive *z*-score indicates spatially clustering of high and/or low health inequality of older people in China, while a negative *z*-score indicates spatially dispersion of high values and low values of health inequality of older people in China.

To complement the global spatial statistics of Moran’s I, local spatial statistics, the Getis-Ord Gi*, is used to evaluate where the clustering or dispersion tested by global Moran’s I is located. In addition, when the spatial clustering is indicated by global Moran’s I, Getis-Ord Gi* can also tell if the clustering is a clustering of high-value or low-value. Mathematically,

(3) $$G_{i}^{*}=\frac{\sum_{j=1}^{n}W_{i,j}x_{j}-\bar{X}\sum_{j=1}^{n}w_{i,j}}{\sum_{j}^{n}W_{i,j}x_{j}}$$

where $W_{i,j}$ is the spatial weight between feature i and j. Positive $G_{i}^{*}$ value shows that high values cluster around i, hence the region is termed “hot spot”; negative value of $G_{i}^{*}$ shows that low values cluster around i, hence the region is termed “cold spot”.

## 3. Results

**Figure 1:**
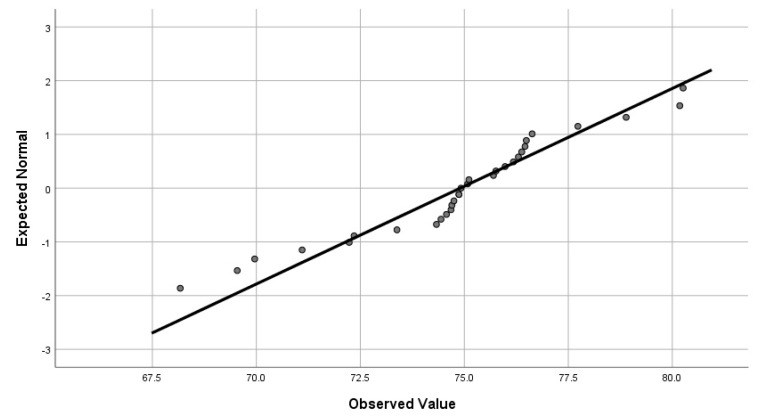
Normal Q–Q plot of life expectancy.

The description of variables (Table 2) in this research indicates that all variables are normally distributed. Figure 1 illustrates that the variable life expectancy is normally distributed. 

**Figure 2:**
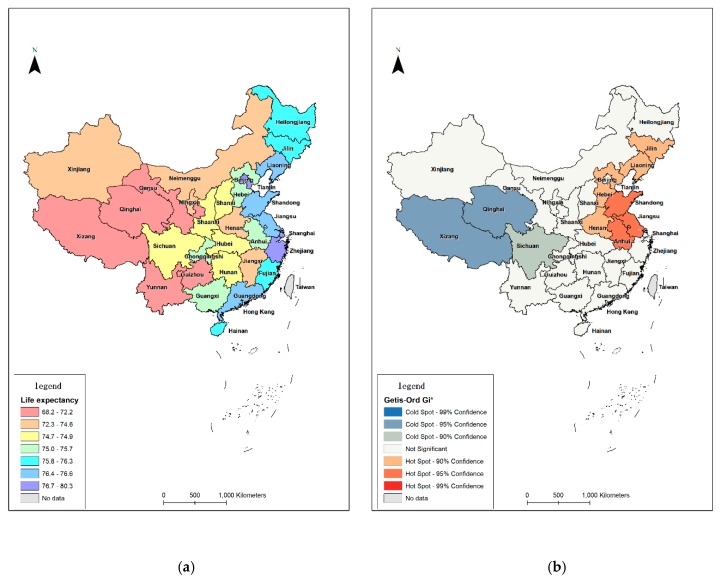
Spatial distribution and clustering of Life expectancy of older people in China (**a**) life expectancy of older people in China, and (**b**) Getis-Ord Gi* of older peple in China.

Table 3 summarizes the values of the dependent variable (life expectancy) and Figure 2a illustrates the spatial distribution of life expectancy of older people in China. We input all the variables (Table 2) into the software SPSS Vision 22 to check their distribution.

Life expectancy in the provinces from the east of China is obviously longer than that in the central and western provinces. The province with the longest life expectancy is Shanghai (80.26 years), followed by Beijing (80.18 years) and Tianjin (78.89 years). On the contrary, provinces with shorter life expectancy are mainly clustered in west China, which are the developing areas. The three provinces with the shortest life expectancy are Xizang (68.17 years), Yunnan (69.54 years), and Qinghai (69.96 years). Figure 2a shows the spatial distribution of life expectancy in China at the national level.

Correlation analysis (Table 4) is used to describe the strength and direction of a linear relationship between two variables. In this research, correlation analysis is conducted with the dependent variable and each independent variable separately. The correlation coefficient, significant level, and cases number are showed in Table 4.

Results of the correlation analysis (Table 4) indicate negative correlations between older people’s life expectancy and percentage of people live on minimum living allowances, percentage of divorced and widowed, and percentage of health centres. According to Cohen’s [25] interpretation of the correlation coefficient (the strength of correlation of r = 0.10 to 0.29 is small; r = 0.30 to 0.49 is medium; r = 0.50 to 1.0 is large), results suggest strong relationships between life expectancy and the percentage of older old-people, gender variables (i.e., percentage of older males and percentage of older females), income variables (i.e., income inequalities, percentage of people live on minimum living allowances and old dependency ratio), living arrangement variable (i.e., ratio between one generation and more), the percentage of health care centres, and the percentage of non-agriculture *Hukou*. A weak relationship is found between life expectancy and the educational variable (i.e., percentage of illiterate population).

The *p*-value of Moran’s I is 0.0001, less than 0.01, indicating that the result of Moran’s I is statistically significant at a 99 percent confidence level, meaning that the life expectancy of older people in China is not randomly distributed. The *z*-score is positive and larger than the *z*-score at 99 percent confidence level of 2.58, indicating that life expectancy of older people in China is clustered, i.e., areas with both high and low life expectancy tend to be close together, respectively. The value of Moran’s I is positive, indicating that the value of life expectancy is positively correlated (Table 5). 

While global Moran’s I shows the general trend, Getis-Ord Gi* shows the spatial pattern in local neighborhoods. The result of Getis-Ord Gi* showed multiple areas of statistically significant high–high clustering in the eastern China (Shandong, Jiangsu, and Anhui provinces) and low–low clustering in western China (Xizang and Qinghai provinces). Shanghai and Beijing are the high–high outliers, indicating that life expectancy in Shanghai and Beijing are low while in the surrounding provinces are high. There is no high–low outlier. For the other provinces, no significant correlation can be detected. Figure 2b shows that there are both high significant hot spots and cold spots for life expectancy for older people at the national level. The hot spots indicate that older people with longer life expectancy are mainly concentrated in Shandong, Jiangsu, and Anhui provinces, while older people with shorter life expectancy are more likely to distributed in the cold spot provinces (Xizang and Qinghai provinces). For the same reason, older people in the medium significant hot spots have shorter life expectancy than those in the most significant hot spots. Older people in the medium significant cold spots have longer life expectancy than those in the most significant cold spots.

## 4. Discussion

The spatial distribution of life expectancy of older people (Figure 2a) indicates health inequalities exist at the national scale in China. An increasing tendency of health inequalities can be seen from east coastal provinces to west remote provinces in China. The spatial distribution of health inequalities corresponds to the variant economic development between eastern and western China.

From the descriptive analysis, there are statistically significant relationships between health inequalities of older people and health-related variables in China. Correlation coefficients are used to measure the strength of the relationship between two variables. Results show that strong positive correlations can be found between life expectancy and the percentage of older old-people as well as gender differences. Being consistent with sub-hypotheses, the more older people, the longer the life expectancy. The Chinese demographic policy, the “one child” policy, as a source of unintended consequences for population aging, contributes to the increase of life expectancy in China. Besides demographic changes, older people’s income status and the living arrangements also have strong relationships with their life expectancy. The negative strong relationship between life expectancy and percentage of people who have minimum living standards indicates that increasing income inequality affects the health outcomes of older people, and the impact of lower income is even worse on older people’s life expectancy. Economic reform results in an increase of income, which is unevenly distributed, corresponding to the growing tendency of life expectancy of older people in China. A strong relationship was found between life expectancy and the *Hukou* status, which is consistent with the hypothesis that people with urban *Hukou* report better health outcomes than rural *Hukou* people. A medium correlation between life expectancy and older people’s marriage status was found. As reported in the hypothesis, being married has a positive influence on older people’s life expectancy, while being divorced or widowed is negatively related to health outcomes of older people. 

With respect to health care accessibility, all variables were correlated with older people’s life expectancy. Older people with better health care resource are more likely to report better health, demonstrating the hypothesis that a better health environment helps to narrow health inequalities.

Consistent with the literature review, the descriptive analysis suggests that variables selected in this study have influence on older people’s life expectancy. Contrary to hypotheses, results indicate that older people’s education level and their marriage status do not have significant influence on their life expectancy. A more accurate description of the effect of educational and marital variables is needed for further research.

The database for this research did not cover all of the provinces. The excluded provinces are Hong Kong, Macau, and Taiwan. These provinces are the well-developed provinces and have the best health outcomes in China. The excluded data might be a problem in generalizing results from this research. 

According to the Getis-Ord Gi* analysis of life expectancy of older people in China, it is interesting to notice that Shanghai and Beijing, with the longest and the second-longest life expectancy, are neither identified as the hot spots nor in cold spot spots. It is possible that Getis-Ord Gi* examines each feature in the neighborhood environment. To be a hotspot with statistical significance, a feature will have high value and be surrounded by other features having high values. The local sum of life expectance in a province is compared proportionally to the sum of all provinces; when the local sum of life expectance is so different from the expected local sum, and when that difference is significantly large so that random chance can be ruled out, we say that the local life expectance is a hot/cold spot. This finding can address more discussion with respect to socio-economic or human perspectives.

## 5. Conclusions

To study the determinants of health inequalities of older people in China and provide a context for the analyses in Section 4 and 5, a descriptive analysis was carried out. Data are measured at the provincial level. All variables reflect older people’s demographic, SES, the health care accessibility and geographical characteristics in China. As predicted, older people’ financial situations, marital status, health care assistance, and *Hukou* status play significant roles in determining older people’s health outcomes. Older people’s educational level impacts life expectancy differentially, as predicted. It could be because that most older people did not receive good education in their youth.

There is a spatial distribution of health outcomes for older people at the national level. Provinces with longer life expectancy are mainly clustered in east China, on the contrary, provinces with shorter life expectancy are mainly clustered in west China. Older people from the coast areas (e.g., Shandong, Jiangsu, Zhejian, Guangdong) often have better health status than people from plateaus (e.g., Xizang Qinhai, Sichuan). The spatial distribution of health inequalities for older people corresponds to the spatial distribution of economic development in China. In terms of life expectancy, result shows an uneven spatial distribution among older people in China. The east coastal provinces have better health outcomes than western remote provinces. How the geographical pattern of health inequalities works among individuals at different geographic scales needs to be investigated at finer levels.

Based on the Getis-Ord Gi* analysis of healthy life expectancy at the national level, Shanghai and Beijing, with the longest life expectancy, are not considered as hot spots. The reason is that there are socio-economic differences between the mega-cities (provinces) and the neighboring provinces. There is a positive correlation between socio-economic status and health status. The better the socio-economic status, the better the health status. Shanghai and Beijing, as the most developed cities, therefore have the best health status in China. Due to the Matthew effect, mega-cities receive an disproportionate amount of health resources, while the neighboring areas can only receive a relatively small amount of resources. The consequence is that neighbouring areas cannot develop as well as mega-cities, and life expectancy in those areas cannot be long enough as in mega-cities. Regional imbalanced development could result in health inequalities. In order to eliminate health inequalities at the national level, it is necessary to eliminate socio-economic differences among provinces. This research contributes an overview of Chinese health inequalities for older people; it demonstrates that the demographic and economic policies have an influence on the increase of life expectancy and the uneven distribution of health outcomes of older people. For further research and investigations should be conducted among individuals at micro-geographic scales.

## Figures and Tables

**Table 1:** Description of variables.

Dependent Variable	Variables
Health inequalities	Life expectancy
Independent variables	Variables
Demographic characteristics	Percentage of older people (ratio between older population and the whole population)
	Percentage of younger old (ratio between younger old and older population)
	Percentage of older old (ratio between older old and older population)
	Percentage of male older (ratio between male older and older population)
	Percentage of female older (ratio between female older and older population)
SES characteristics	Income inequalities (ratio between older people with high income and low income)
	Percentage of people live on minimum living allowances (ratio between older people live on minimum living allowances and older population)
	Old dependency ratio (ratio between the number of older people and working-age population)
	Percentage of illiterate population (ration between older people who cannot read and older population)
	Percentage of divorced and widowed (ratio between divorced or widowed older people and older population)
	Percentage of people with married status (ratio between married older people and older population)
	Ratio between one person and more (ratio between older people live alone and live with more than one person)
	Ratio between one generation and more (ratio between older people live with spouse and older people live with more than one generation)
Health care accessibility	Percentage of hospitals (ratio between the number of hospitals in each province to hospitals in China)
	Percentage of health care centers (ratio between the number of health care centers in one province to health care centers in China)
	Health care centers per 1000 population (ratio between health care centers in each province to 1000)
Geographic characteristics	Percentage of non-agriculture *Hukou* (ratio between people with non-agriculture *Hukou* to agriculture *Hukou* in each province).

All variables selected from the three datasets, reflecting characteristics of older people (i.e., people age 60 years old and over); National surveys cover 31 provinces, autonomous regions, and municipalities that are directly under the Central Government in China, excluding Hong Kong, Macau, and Taiwan.

**Table 2:** Description of variables.

Variable	Mean	Median	Min	Max	Range
Life expectancy	74.90	74.92	68.17	80.26	12.09
Percentage of older people	12.64	12.75	6.94	17.56	10.62
Percentage of younger old	11.30	11.59	6.91	15.43	8.52
Percentage of older old	1.46	1.45	0.67	2.55	1.89
Percentage of male older	6.26	6.30	3.48	8.80	5.32
Percentage of female older	6.50	6.63	4.19	8.62	4.42
Income inequalities	1.36	1.09	0.48	6.20	5.72
Percentage of people live on minimum living allowances	4.47	3.98	2.12	9.25	7.12
Old dependency ratio	12.82	12.39	9.23	17.97	8.74
Percentage of illiterate population	0.72	0.72	0.67	0.78	0.11
Percentage of divorced and widowed	7.16	6.99	4.96	9.39	4.43
Percentage of people with married status	71.48	72.11	57.45	75.69	18.24
Ratio between one person and more	0.17	0.15	0.10	0.33	0.23
Ratio between one generation and more	0.53	0.52	0.29	1.02	0.72
Percentage of hospitals	2.81	2.34	1.39	6.64	5.25
Percentage of health centres	4.83	4.63	0.00	13.25	13.25
Health care centres per 1000 population	3.30	2.99	2.22	6.80	4.58
Percentage of non-agriculture *Hukou*	31.93	28.08	14.77	61.89	47.12

*N* = 31.

**Table 3:** Table of dependent variable.

Region	Region NO.	Life Expectancy (Years)	Rank
Anhui	1	75.08	18
Beijing	2	80.18	30
Chongqing	3	75.70	17
Fujian	4	75.76	21
Gansu	5	72.23	6
Guangdong	6	76.49	24
Guangxi	7	75.11	14
Guizhou	8	71.10	3
Hainan	9	76.30	22
Hebei	10	74.87	20
Heilongjiang	11	75.98	19
Henan	12	74.57	15
Hubei	13	74.87	12
Hunan	14	74.70	11
Inner Mongolia	15	74.44	8
Jiangsu	16	76.63	26
Jiangxi	17	74.33	7
Jilin	18	76.18	23
Liaoning	19	76.38	25
Ningxia	20	73.38	10
Qinghai	21	69.96	4
Shaanxi	22	74.68	9
Shandong	23	76.46	27
Shanghai	24	80.26	31
Shanxi	25	74.92	16
Sichuan	26	74.75	13
Tianjin	27	78.89	29
Xizang	28	68.17	1
Xinjiang	29	72.35	5
Yunnan	30	69.54	2
Zhejiang	31	77.73	28

*N* = 31; rank sorts from the smallest to largest.

**Table 4:** Correlation of variables.

Variables	Correlation Coefficient (r)	Sig (2-tailed)
Life expectancy	1.000	
Percentage of older people	0.499 **	0.004 **
Percentage of younger old	0.453 *	0.010 *
Percentage of older old	0.744 **	0.000 ***
Percentage of male older	0.500 **	0.004 **
Percentage of female older	0.567 **	0.001 **
Income inequalities	0.605 **	0.000 ***
Percentage of people live on minimum living allowances	−0.597 **	0.000 ***
Old dependency ratio	0.532**	0.002 **
Percentage of illiterate population	0.163	0.382
Percentage of divorced and widowed	−0.429 *	0.016 *
Percentage of people with married status	0.322	0.077
Ratio between one person and more	0.411 *	0.021 *
Ratio between one generation and more	0.702 **	0.000 ***
Percentage of hospitals	0.412 *	0.021 *
Percentage of health centres	−0.702 **	0.000 ***
Health care centres per 1000 population	0.477 **	0.007 **
Percentage of non-agriculture *Huko*u	0.604 **	0.000 ***

Dependent variable: life expectancy; *N* = 31; sig. * *p* < 0.05; ** *p* < 0.01; *** *p* < 0.001. **. Correlation is significant at the 0.01 level (2-tailed); * Correlation is significant at the 0.05 level (2-tailed).

**Table 5:** Moran’s I of life expectancy of older people in China.

Moran’s I	0.266
*Z*-score	3.906
*P*-value	0.0001 ****

**** *p* < 0.0001
